# Captive bottlenose dolphins and killer whales harbor a species-specific skin microbiota that varies among individuals

**DOI:** 10.1038/s41598-017-15220-z

**Published:** 2017-11-10

**Authors:** M. Chiarello, S. Villéger, C. Bouvier, J. C. Auguet, T. Bouvier

**Affiliations:** Marine Biodiversity, Exploitation and Conservation, Université de Montpellier, CNRS, IFREMER, IRD, Montpellier, France

## Abstract

Marine animals surfaces host diverse microbial communities, which play major roles for host’s health. Most inventories of marine animal surface microbiota have focused on corals and fishes, while cetaceans remain overlooked. The few studies focused on wild cetaceans, making difficult to distinguish intrinsic inter- and/or intraspecific variability in skin microbiota from environmental effects. We used high-throughput sequencing to assess the skin microbiota from 4 body zones of 8 bottlenose dolphins (*Tursiops truncatus*) and killer whales (*Orcinus orca*), housed in captivity (Marineland park, France). Overall, cetacean skin microbiota is more diverse than planktonic communities and is dominated by different phylogenetic lineages and functions. In addition, the two cetacean species host different skin microbiotas. Within each species, variability was higher between individuals than between body parts, suggesting a high individuality of cetacean skin microbiota. Overall, the skin microbiota of the assessed cetaceans related more to the humpback whale and fishes’ than to microbiotas of terrestrial mammals.

## Introduction

Marine animals’ surfaces are associated with highly diverse microbial communities, which play major roles for their health, including protection against macrofouling, and pathogens^1,2^. These surface microbiota were shown to be both distinct from surrounding planktonic samples^1^, and host-species specific^2^, suggesting that they could have coevolved with their animal hosts^3^. In addition, marine animal surface microbiota are dynamic assemblages^4^, with composition of microbial Operational Taxonomic Units (OTUs) as well as their relative abundance varying between host life stages^5^, surrounding environmental conditions^6^ and geographical location^7^. However, most of these findings have been reported from marine invertebrates, and especially corals. Whether these observations could be generalized to marine vertebrates, which constitute the most important biomass fraction of macroorganisms in the global ocean, is barely unknown (but see recent work on fishes^8,9^ and whales^10^). Among marine vertebrates, mammals are represented by more than 100 species belonging to three clades (pinnipeds, cetaceans and sirenians) which respective ancestors were terrestrial. Marine mammals hence have biological features, including skin structure, similar to terrestrial mammals. Therefore, assessing the composition of skin microbiota of marine mammals could shed light on the importance of evolutionary legacies and adaptation to marine environment in shaping skin microbiota of animals.

The only marine mammal skin microbiota described to date is the one of the free-ranging humpback whale from the North Pacific. Apprill *et al*.^10,11^ showed that individuals share a core skin microbiota and that variability in taxonomic and phylogenetic diversity of skin microbiota among individuals is driven by geographical location and the health state of the whale. However, such studies on wild animals do not allow disentangling individual-driven variation of skin microbiota from the effect of environmental conditions. Animals housed in controlled environment offer the opportunity to measure the interspecific and inter-individual variability of animals skin microbiota independently from environmental variability, and to assess the intra-individual variability of their microbiota^12^.

Besides assessing the taxonomic and phylogenetic facets of skin microbiota, describing its functional role is fundamental to understand the link between microbiota and host health. Indeed, skin is the first line of defense from pathogen infections in mammals with skin microbiota closely interacting with its host cells from the epidermis to the deep dermis^13^, to modulate immunity^14,15^, and support antagonistic effects against pathogens^16^. However the functional diversity of the skin microbiota of marine mammals has never been assessed, as well as its congruence with its phylogenetic diversity^17^.

Recent advances in bioinformatics (e.g. PICRUSt^18^) allow predicting metagenome functional content from 16 S rDNA data and hence to assess simultaneously the taxonomic, phylogenetic and potential functional diversities of microbial communities.

Here, using high-throughput sequencing, we assessed the taxonomic and phylogenetic diversities of the skin microbiota from 4 body zones (*i.e*. the dorsal, anal and pectoral fins, and its anal zone) of 8 individuals of two emblematic Odontoceti (toothed whales) species, the bottlenose dolphin (*Tursiops truncatus*) and the killer whale (*Orcinus orca*), housed in controlled conditions. We also predicted the functional facet of microbiota diversity using PICRUST software. We first measured the similarity between the microbiota of the two species. Second, we quantified the magnitude of intraspecific variability of microbiota, *i.e*. between individuals of each species and between their body parts. Third, we analyzed the similarity between the skin microbiota of cetaceans and those of terrestrial mammals and non-mammal vertebrates.

## Material and Methods

### Sampling of skin and planktonic microbiotas

We sampled skin microbiota of four killer whales (*Orcinus orca*) and four bottlenose dolphins (*Tursiops truncatus*) housed at Marineland park (Antibes, France) in accordance with European laws (Directive EC 1999/22 and EU CITES 338/97). Animals were manipulated by their caretakers, in accordance with internal practices of the park. Sampling was done using a non-invasive method (swabbing a small surface for 1 minute). All manipulations were approved by Marineland’s scientific committee.

Killer whales and dolphins were aged from 13 to more than 30 years at the time of sampling (Table 1). Contrary to dolphins, killer whales were affiliated, with the younger ones being siblings or half-siblings, and the older one (Freya) being the mother of the older male (Valentin). All animals but one (*i.e*. Valentin which received an antifungal treatment that ended two weeks before the day of sampling) did not receive any antibiotics during the 6 months before sampling.

**Table 1:** Animals included in this study.

Species	Individual	Age (years)	Sex	Complementary Information
**K. whales**	Freya	>30	Female	*Valentin’s mother*
	Valentin	18	Male	*Antifungal treatment ended 15 days before sampling*
	Wikie	13	Female	*Valentin’s half sister, sister of Inouk*
	Inouk	15	Male	*Valentin’s half brother, brother of Wikie*
**Dolphins**	Sharki	>30	Female	
	Lotty	>30	Female	
	Dam	17	Male	
	Rocky	15	Male	

The age of each animal at sampling time is indicated, as well as their kinship, when known. Animals older than 30 years old were captured from the wild during the early 1980s; therefore their exact age is unknown.

Individuals of the two species were kept in two separated pools, which are filled by the same seawater circulation system. Seawater is pumped from 600-meters offshore and 68-meters deep in the Mediterranean Sea, and filtered through sand. Water flux is set so that the water of each pool is renewed every 2 hours.

The day of sampling, each animal was asked by its caretaker to raise successively 4 body zones (*i.e*. the dorsal, caudal and pectoral fins, and anal zone) outside of water. These four zones could be considered as distinct patches for microbiotas (*i.e*. distant to each other by >30 cm) and experience different micro-environmental conditions (*e.g*. the anal zone because of release of feces and urine). After briefly rinsing the skin using 100-mL autoclaved seawater, skin microbiota was sampled by swabbing a 63-cm^2^ circular surface using sterile foam-tipped applicators from Whatman (GE Healthcare) during 30 seconds on each side of the swab. For the caudal and pectoral fins, only the upper side of the fin was sampled. We then cut the tip of the swab using ethanol-rinsed scissors and placed the sponge part of the swab into sterile cryotubes.

For each species, three 100-mL pool and input water (*i.e*. exit of pipe from filtering system) samples were collected and filtrated through a 47 mm diameter, 0.2 µm pore size, polycarbonate membrane (Whatman, Clifton, USA). The membranes were then placed in sterile cryotubes. All samples were immediately snap-frozen in liquid nitrogen, transported to the laboratory and stored at −80 °C before DNA extraction.

### 16S rDNA amplification and sequencing

DNA was extracted using the DNeasy Blood & Tissue kit (Qiagen, ID 69504) following the manufacturer’s protocol with a few modifications. Briefly, swabs were placed in 2 mL sterile microtubes, and 260 µL of enzymatic lysis buffer were added. After a 30-minutes incubation at 37 °C, 50 µL of proteinase K and 200 µL of AL buffer were added before the incubation at 56 °C for 30 minutes. The elution step was done twice in 100 µL of elution buffer. The two eluates were pooled to obtain a single 200 µL DNA sample per swab. DNA quality and quantity was assessed by spectrophotometry (NanoDrop 1000, Thermo Fisher Scientific, USA).

The V3-V4 region of the 16 S rDNA gene was amplified using bacterial primers modified for Illumina sequencing 341 F (5′-CTTTCCCTACACGACGCTCTTCCGATCT-ACGGRAGGCAGCAG- 3′)^19^ and 784 R (5′ - GGAGTTCAGACGTGTGCTCTTCCGATCT-TACCAGGGTATCTAATCCT- 3′)^20^. Amplification was very difficult due to the low DNA concentration, and possible contamination by keratinocytes in skin samples. Consequently skin and water samples were amplified using two different PCR kits and conditions, which are provided in Supplementary Information S1. Both sample types were amplified in triplicates. After PCR, the success of amplification was verified by migration on agarose gels, and equal volumes of three PCR products were pooled for each sample. After pooling, final concentration measured by Nanodrop (Wilmington, USA) averaged 14 ng. µL^−1^ (±17, *n* = 43). After amplification, equimolar amounts of all PCR products were pooled and cleaned up using calibrated Ampure XP beads by an external laboratory (MR DNA, Shallowater, USA) and sequenced on a single run of Illumina platform using the 2 × 250 bp MiSeq chemistry. To check biases induced by the two different PCR protocols, we amplified 2 water DNA samples using both PCR kits and compared them after sequencing. They showed similar community structure (see S1). The nucleotide sequence data is available in the NCBI SRA database under the biosample numbers SAMN07278850-SAMN07278894.

### Sequence processing and phylogenetic analyses

Assembly of paired reads was performed by the sequencing platform. All subsequent steps of sequence processing were performed following the SOP of Kozich *et al*. for MiSeq.^21^, https://www.mothur.org/wiki/MiSeq_SOP, 2016) using Mothur^22^. After removing sequences with an irregular length (*i.e*. outside a range of 420–460 pb), sequences were aligned along the SILVA reference database^23^ (release 123). Unaligned sequences were removed from the final alignment during this process. Chimeras were removed using UCHIME^24^. Filtered sequences were then classified using the SILVA reference taxonomy and the non-bacterial reads were removed. After these steps, we obtained a total of 2,198,758 sequences from our 43 samples, with 51,133 ± 20,883 (expressed as Mean ± SD) sequences per sample. The number of sequences read for each sample is unlikely correlated with total abundance of bacteria in sample, while it could bias assessments of microbial biodiversity. Therefore, to ensure that further diversity assessments were not biased by the uneven sequencing efficiency among samples, 10,000 sequences were sub-sampled within each sample (Supplementary Information S2). Non-parametric Chao’s coverage estimator was computed in each community to assess effect of subsampling level using “Coverage” function provided in *entropart* R-package^25^. This index averaged 0.98 ± 0.008 among microbial communities testifying for the accuracy of further diversity analyzes.

Sequences were then clustered into OTUs with 99% sequence identity, and the dominant sequence for each OTU was selected as reference and aligned against the SILVA reference database using Mothur for subsequent phylogenetic tree reconstruction. An outgroup was defined using a set of archaeal sequences obtained from SILVA database and re-aligned against the previous alignment of reference sequences using the MAFFT v7 with –add option^26^ before tree reconstruction using Fasttree^27^.

To estimate the potential functions of microbial OTUs based on 16 S rDNA data, we used PICRUST software^18^ on reference sequences, using KEGG orthologs^28^ grouped into pathways (function *categorize_by_function.py*, *level* = 3). A matrix containing 329 pathways was obtained. We then removed all eukaryotic functions, for instance genes related to cardiovascular diseases and categories grouped as “organismal systems”. NSTI values averaged 0.04 ± 0.02 and 0.12 ± 0.02 respectively in skin-associated and planktonic communities, indicating that OTUs sequences were close enough to the nearest 16 S rDNA of reference genomes to infer functions.

### Investigating the presence of pathogens

Two additional phylogenetic analyses were performed separately for the two genera *Staphylococcus* and *Streptococcus* to look for putative pathogenic bacteria on cetacean skin. Near full-length 16 S rDNA sequences of well-known pathogenic and non-pathogenic species of these genera were downloaded from the SILVA database (ACC number provided in Supplementary S3). Reference sequences of the most abundant OTUs belonging to these two genera (*i.e*. 35 Staphylococci and 31 Streptococci sequences), as well as the SILVA sequences were aligned against the SILVA reference database using Mothur, and added into the SILVA reference phylogenetic tree using ARB software^29^. The full phylogenetic tree was then pruned using the *ape* R-package^30^ to remove all but the added sequences, while keeping the topology of the tree. We then visualized the phylogenetic tree to determine if OTUs from this study were close to the pathogenic species considered.

### Assessing diversity of and dissimilarity between skin microbiotas

Four complementary diversity indices were computed to assess the taxonomic and phylogenetic facets of diversity, including their respective compositional and structural components^9^.

The compositional diversity accounts only for the presence/absence of OTUs or phylogenetic lineages (here defined as subsets of the phylogenetic tree, containing OTUs and their associated branch lengths). Compositional taxonomic diversity was measured by counting the number of OTUs in a sample (OTUs or functional richness). The phylogenetic compositional diversity (*i.e*. the phylogenetic richness) was measured as Faith’s PD^31^ divided by the total PD of the tree (to scale values between 0 and 1). The structural diversity accounts for the relative abundance of OTUs or phylogenetic lineages, based on the number of sequences represented by each OTU.

The taxonomic structural diversity was computed using the Shannon index^32^, expressed in Hill numbers^33^ on abundance of OTUs. The phylogenetic structural diversity was measured using the Allen index^34^. All diversity indices were computed using R software. The taxonomic alpha diversity indices were computed using our own functions (available at https://github.com/marlenec/chao), while the Faith PD and Allen index were calculated respectively using the *picante* and *entropart* packages^25,35^.

Similarly, we used four complementary beta-diversity indices to assess the taxonomic and phylogenetic dissimilarity between pairs of microbiotas, according to their composition or structure. The compositional taxonomic dissimilarity was assessed based on presence/absence of OTUs, using the Sorensen index^36^ computed with *betapart* package^37^. The structural taxonomic dissimilarity, taking into account the relative abundance of OTUs, was measured using the multiplicative decomposition of the Shannon index^9^. The phylogenetic compositional and structural dissimilarities were computed using the unweighted and weighted versions of the Unifrac index^38,39^, respectively, from the *GUniFrac* package^40^.

Kruskal-Wallis tests (KW) were performed on alpha-diversity indices to assess the effect of sample type (*i.e*. water vs. skin samples), species, individual, sex, or body zone on microbial alpha-diversity. When significant, the KW was followed by post-hoc pairwise comparisons among groups using the *pgirmess* package, which includes the correction for multiple tests from Siegel and Castellan^41,42^. The correlation between the age of the individual and its associated alpha-diversity was assessed using a Spearman’s correlation test using *stats* R-package. Beta-diversity values were visualized on PCoA plots using the *ape* package^30^. The effect of sample type, species, individual, age, sex, and body zone on the structure and composition of microbial communities was assessed by performing separated one-factor PERMANOVAs with 999 permutations on beta-diversity values using *vegan* package^43^. The number of identical OTUs between skin microbiota and planktonic communities was analyzed using an Euler Diagramm computed with *venneuler* R-package^44^. To assess how each microbial clade contributed to the dissimilarity between planktonic and skin microbiotas, as well as between microbiotas of cetacean species, we performed a LefSe analysis^45^. LefSe provides Linear Discriminant Analysis (LDA) scores for the bacteria clades contributing the most to the differences between cetacean species.

### Comparing skin microbiota of cetaceans and other vertebrates

The skin microbiota of dolphins and killer whales was compared to the published skin microbiota of 11 terrestrial and marine vertebrates, namely Human^46–48^, pig^49^, humpback whale^10^ and eight teleostean fish species^8,9^. Due to the different primers that were used for these different species, we could not directly reanalyze sequences from studies to assess OTUs abundance. Therefore, we extracted clades relative abundance from published figures and averaged across all individuals (*i.e*. 36 humans, 4 pigs and 57 humpback whales) for each mammalian species. In the case of marine fishes, as individual data was not available for all species, we chose to average clades relative abundances of all species to make a single “fish” category. The most abundant clades colonizing the animals were averaged for each animal; and a Bray-Curtis dissimilarity index (BC)^50^ between the different microbiotas was computed based on the relative abundance of the different clades. A BC index of 1 indicates that microbiotas are maximally dissimilar, *i.e*. that they are dominated by different clades while a BC = 0 indicates that the two microbiotas have the same taxonomic structure (*i.e*. same clades with same abundances).

## Results

### Diversity of skin and planktonic microbiotas

**Figure 1:**
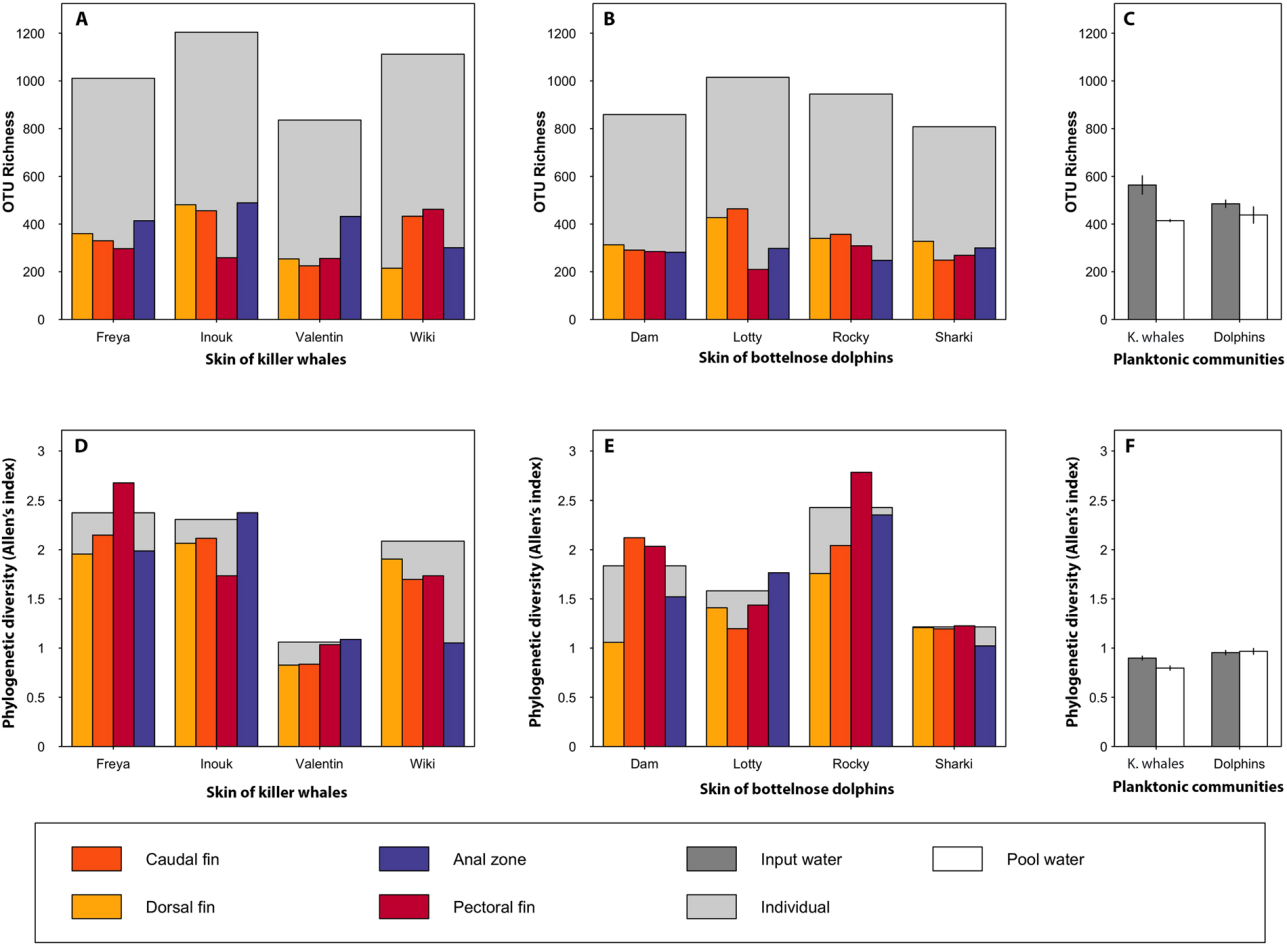
Biodiversity of skin microbial communities from captive killer whales (**A** and **D**) and common bottlenose dolphins (**B** and **E**), and planktonic communities (**C** and **F**). The first row of plots (**A**–**C**) illustrates taxonomic richness, *i.e*. number of OTUs observed in a sample and the second row of plots (**D**–**F**) illustrates phylogenetic diversity, measured using Allen’s index (*i.e*. accounting for relative abundance of phylogenetic lineages). Total diversity of each individual (*i.e*. accounting for all body zones sampled) is illustrated with larger light-gray bars. Bars on panels C and F represent the mean (and associated standard deviation) of OTU richness and phylogenetic diversity for planktonic communities (*n* = 3 water samples). “Pool” refers to animal’s surrounding water, and “Input” refers to the water sampled at the exit of filtering system.

We recovered a total of 7,287 OTUs among our 43 samples, with OTU richness ranging from 210 to 606 across samples. Water samples (481 ± 64 OTUs, n = 11 samples) were significantly richer than skin samples (332 ± 84 OTUs, n = 32 samples) (Kruskal-Wallis, P < 0.0001, Fig. 1). However when considering the relative abundance of OTUs, skin samples were significantly more diverse than water samples (KW, P = 0.001), with a Shannon index of 30.4 ± 23.6 and 9.2 ± 2.5 equivalent number of species, respectively (Supplementary Information S4). Phylogenetic richness did not significantly differ between planktonic and skin-associated communities (KW, P = 0.06, S4). However, when taking into account the relative abundance of phylogenetic lineages, skin samples were significantly more diverse than water samples (KW, P < 0.0001), with Allen index being ca. 1.8 times higher in skin-associated communities than in the planktonic ones (Fig. 1).

Skin-associated microbial alpha-diversity did not significantly differ between species (KW, P > 0.05). At intraspecific level, there was no effect of individuals or body zones on OTUs richness and phylogenetic richness (KW, P > 0.05, Figs 1 and S4). However a significant effect of individual on taxonomic diversity was found for both species (Shannon index, KW, P = 0.02 and 0.01 for dolphins and killer whales, respectively), which was not explained by age (Spearman’s correlation test, P > 0.05) or sex (KW, P > 0.05). Post-hoc pairwise comparisons showed that the dolphin Sharki hosted significantly lower level of taxonomic diversity than Rocky; and that killer whale Valentin hosted significantly lower taxonomic diversity than Freya, Inouk and Wiki (P < 0.05, S4). The microbiotas of these individuals also have contrasted levels of phylogenetic diversity (P < 0.05).

### Dissimilarity between microbiotas

**Figure 2:**
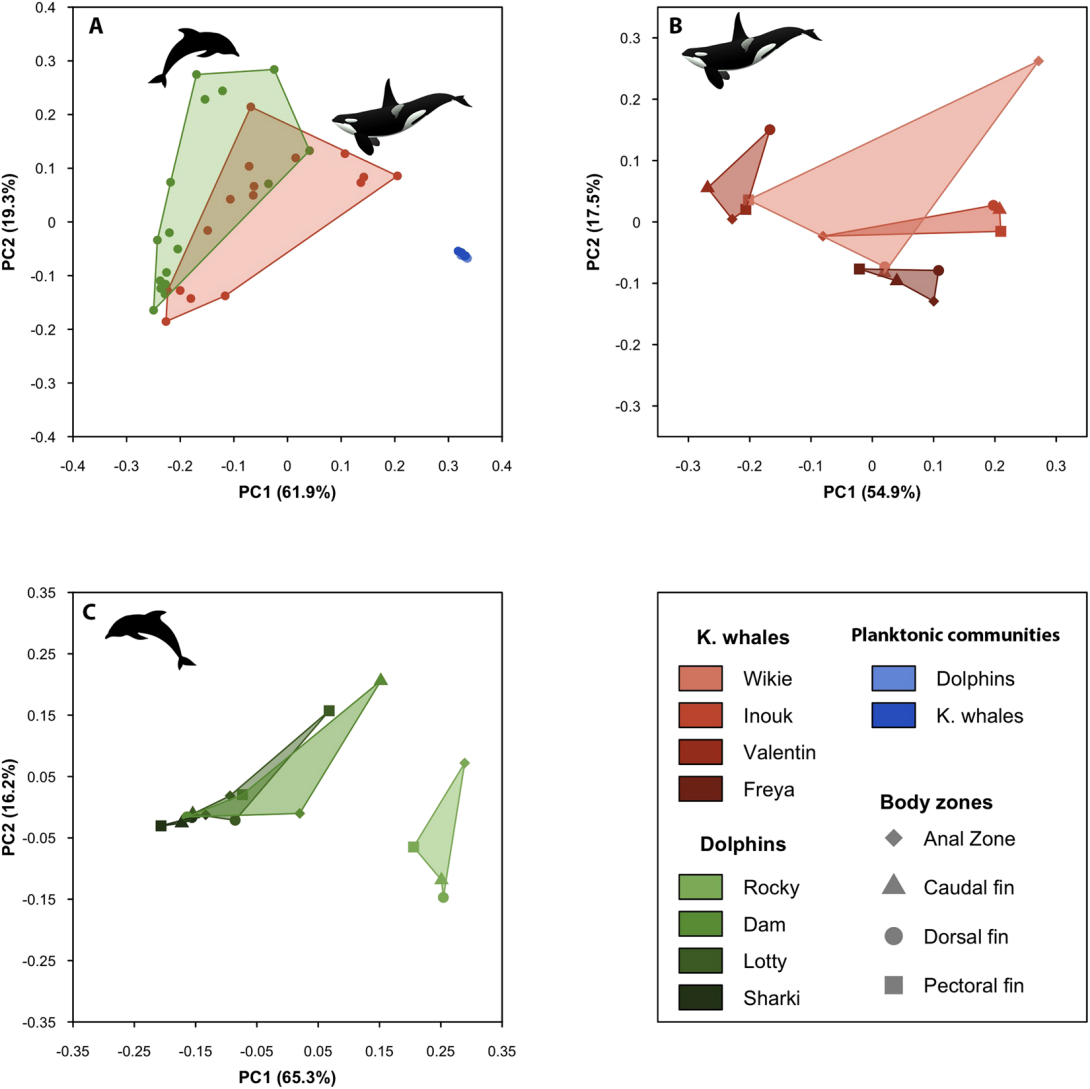
Phylogenetic dissimilarity between microbial communities illustrated along the two first axes from Principal Coordinates Analyses computed on weighted Unifrac dissimilarity values. All samples (*i.e*. skin-associated communities of 4 body zones of 4 captive common bottlenose dolphins and of 4 killer whales, and planktonic communities from respective pools and exit of filtering system) are illustrated on panel (A). Panels (B) and (C) represent only captive killer whales or common bottlenose dolphin skin-associated communities with the 4 body zones of each individuals being delimitated by a polygon.

**Figure 3:**
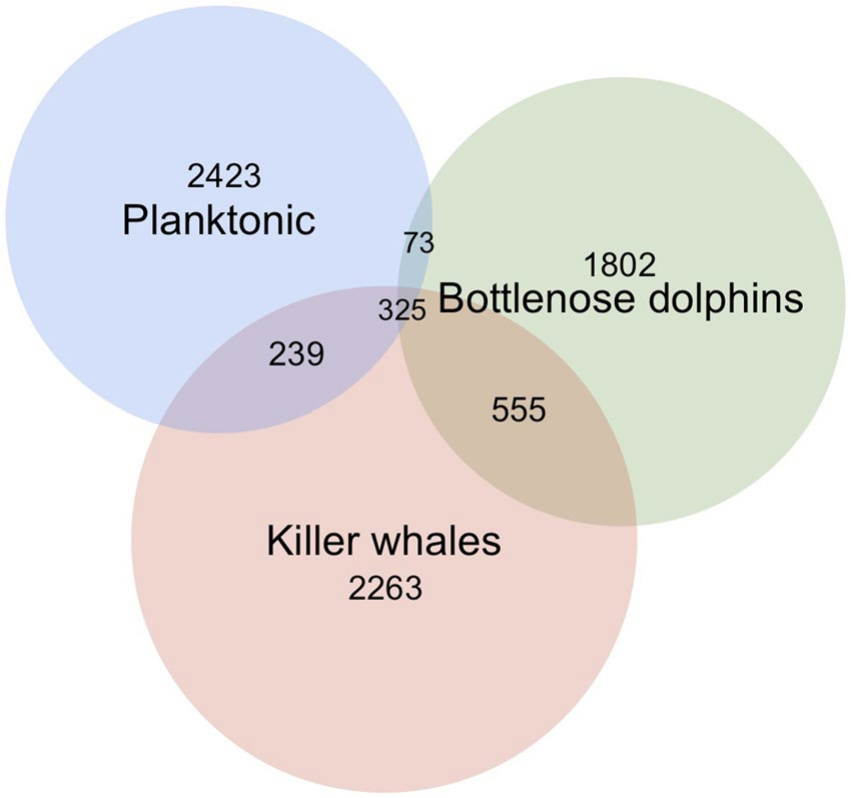
Euler diagram representing the number of skin-associated OTUs from each species (killer whale and common bottlenose dolphin) and planktonic communities that are shared or unique.

Water communities and skin-associated microbial communities significantly differed for all facets of biodiversity considered (Table 2, Fig. 2, Supplementary Information S6). For instance, only 12% of OTUs found on cetacean were also present in surrounding planktonic communities (Fig. 3). Taxonomic (0.61 ± 0.17) and phylogenetic (0.43 ± 0.19) dissimilarities reached their higher level between planktonic and skin-associated communities (S5).

**Table 2:** Determinants of biodiversity of skin microbial communities.

Biodiversity facets	Taxonomic				Phylogenetic			
Dissimilarity indices	Sorensen		Beta-Shannon		U-Unifrac		W-Unifrac	
**Factor**	R^2^	P	R^2^	P	R^2^	P	R^2^	P
Plankton vs. skin	0.09	**0.001**	0.36	**0.001**	0.12	**0.001**	0.46	**0.001**
Dolphins vs. K. whales	0.06	**0.001**	0.14	**0.001**	0.06	**0.001**	0.10	**0.032**
**Killer whales**								
Individuals	0.23	**0.010**	0.32	**0.026**	0.24	**0.018**	0.48	**0.002**
Body zones	0.19	0.683	0.15	0.805	0.20	0.420	0.10	0.957
Age	0.07	0.105	0.07	0.421	0.08	0.116	0.07	0.316
Sex	0.08	0.092	0.09	0.163	0.07	0.311	0.10	0.159
**Dolphins**								
Individuals	0.22	**0.035**	0.51	**0.001**	0.21	0.102	0.60	**0.002**
Body zones	0.20	0.569	0.10	0.961	0.20	0.663	0.08	0.975
Age	0.07	0.211	0.16	**0.044**	0.07	0.234	0.36	**0.003**
Sex	0.07	0.332	0.14	0.068	0.07	0.377	0.31	**0.007**

Effect of each factor was tested using permutational ANOVAs (PERMANOVAS, 999 permutations) on dissimilarity matrices with for each facet of biodiversity, two indices: one index accounting only for composition of OTUs or phylogenetic lineages (*i.e*. Sorensen or Unweighted Unifrac), and one index accounting for relative-abundance of OTUs or phylogenetic lineages (*i.e*. beta-Shannon or weighted Unifrac). Bold P-values (<0.05) indicate a significant effect of the tested factor. Partial R-squared (R^2^) is the proportion of variation in the dissimilarity matrix explained by the tested factor.

Dolphin- and killer whale-associated communities significantly differed for both taxonomic and phylogenetic diversity, as revealed by PERMANOVA (Table 2). Within each species, individuals had skin microbiotas with significantly different phylogenetic structure (Table 2). The age of the individual, and to a lesser extent, its sex, had a significant effect on diversity of dolphin skin microbiota when considering relative abundances of OTUs or phylogenetic lineages (PERMANOVA, Table 2). For killer whales, neither age nor sex had a significant effect on skin-associated microbiota. Microbiotas from the four studied body zones were not significantly different (PERMANOVA, Table 2).

### Composition of bacterial communities

**Figure 4:**
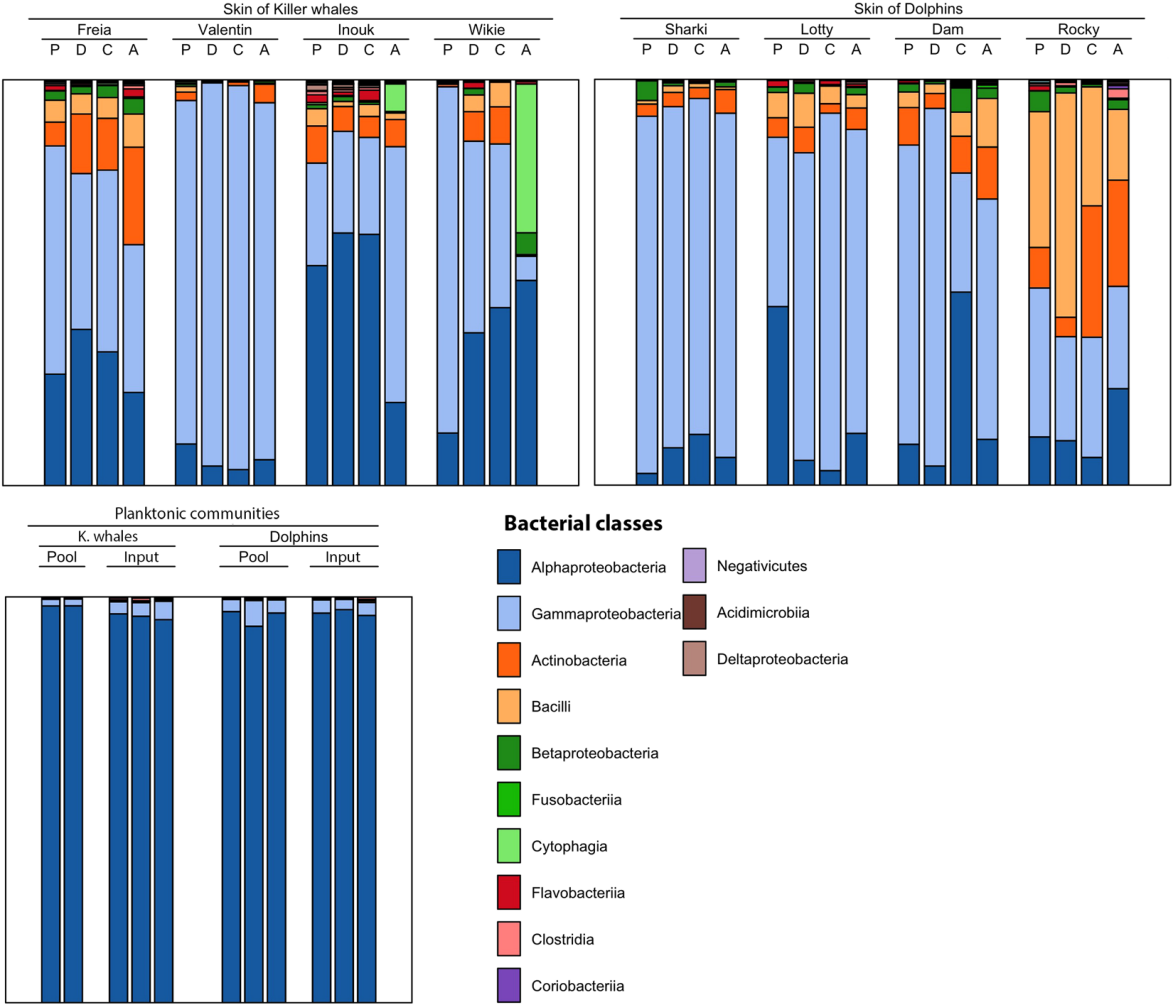
Mean relative abundance of bacterial classes in skin-associated communities of common bottlenose dolphin and killer whales, and planktonic communities. P: upper side of pectoral fin, D: dorsal fin, C: upper side of caudal fin, A: anal zone. “Pool” refers to animal’s surrounding water, and “Input” refers to the water sampled from the exit of pipe from filtering system.

Planktonic communities were dominated by *Alphaproteobacteria* (95.9 ± 0% of sequences), especially *Hyphomonadaceae*, *Rhodospirillaceae* and *Rhodobiaceae* (71.4 ± 0.1%, 7.6 ± 0.1% and 6.6 ± 0.1% of all *Alphaproteobacteria*, respectively) (Figs 4 and S6). Ca. 90% of planktonic OTUs could not be identified at genus level, excepted *Anderseniella sp*. [*Alphaproteobacteria*], which contributed to 6.3 ± 0.1% of sequences in water samples (S6).

Cetacean skin microbiota was mostly composed of *Gammaproteobacteria* (57.5 ± 27%), *Alphaproteobacteria* (22.4 ± 18.8%), *Actinobacteria* (7.9 ± 0.1%) and *Bacilli* (7.3 ± 0.1%) (Fig. 4). The most abundant genus on both species was *Psychrobacter sp*. [*Gammaproteobacteria*], which dominated skin samples, making 30.1 ± 31% of total abundance on killer whales’ skin and 45.2 ± 28% on dolphins’ skin, while they represented a small fraction of sequences (1.1 ± 0.2%) in planktonic communities (S6). Other genera were abundant in a few skin samples, including *Enhydrobacter* (8.4 ± 11.9% in both species), *Staphylococcus* (4.6 ± 7.5%), *Sphingomonas* (3.5 ± 5.9%), *Paracoccus* (2.8 ± 4.9%) and *Gardnerella* (0.4 ± 0.7%) (S6). Families and genera revealed by LefSe analysis for the two host species were mostly scarse and are not visible in S6. Four biomarkers were found for killer whales’ skin, belonging to *Alphaproteobacteria*: *Phyllobacteriaceae* (log10 effect size = 4.5), *Rubellimicrobium* and *Ruegeria* (*Rhodobacteraceae*) (3.6 and 3.5), and *Microvirga* (*Methylobacteriaceae*) (3.4). Three biomarkers were significantly more abundant on dolphin’s skin: *Nocardiaceae* (3.4), *Enterobacteriaceae* (3.2) and *Caulobacter* (*Caulobacteraceae*) (3.1).

Among OTUs from skin microbiotas, 237 were identified as *Staphylococcus sp*., with the most abundant one averaging 4.4% of sequences in dolphin-associated communities. The phylogenetic analysis of the 35 most abundant ones showed that none of them was related to the recognized marine mammal pathogen *Staphylococcus delphini* or other pathogenic staphylococci (Supplementary S3). The most abundant *Staphylococcus* was closely related to the opportunist *Staphyloccocus warneri*. 45 OTUs identified as *Streptococcus* were recovered in skin-associated communities, with the most abundant one averaging 0.5% of sequences of both species’ microbiotas. None of the 40 most abundant OTUs were related to the pathogenic hemolytic *Streptococci*.

### Potential functional diversity of planktonic and skin microbiota

LefSe analysis identified 19 functional biomarkers of planktonic communities, with strongest effect sizes for pathways involved in environmental information processing (Supplementary Information S7). Other functional biomarkers of planktonic communities were pathways related to cellular processes, especially those related to motility (flagellar assembly pathway and motility proteins, being respectively twice and 60% more abundant in planktonic communities) and cell cycle, principally reflected by *Caulobacter* cell cycle pathways.

Cetacean skin microbiota was characterized by functions involved in genetic information processing, especially pathways related to DNA repair and recombination proteins (Supplementary Information S7), DNA replication and translation (especially from 0.9 to 1.3% of proteins involved in ribosome biogenesis in skin communities).

### Comparison of cetacean microbiota with other vertebrate microbiotas

**Figure 5:**
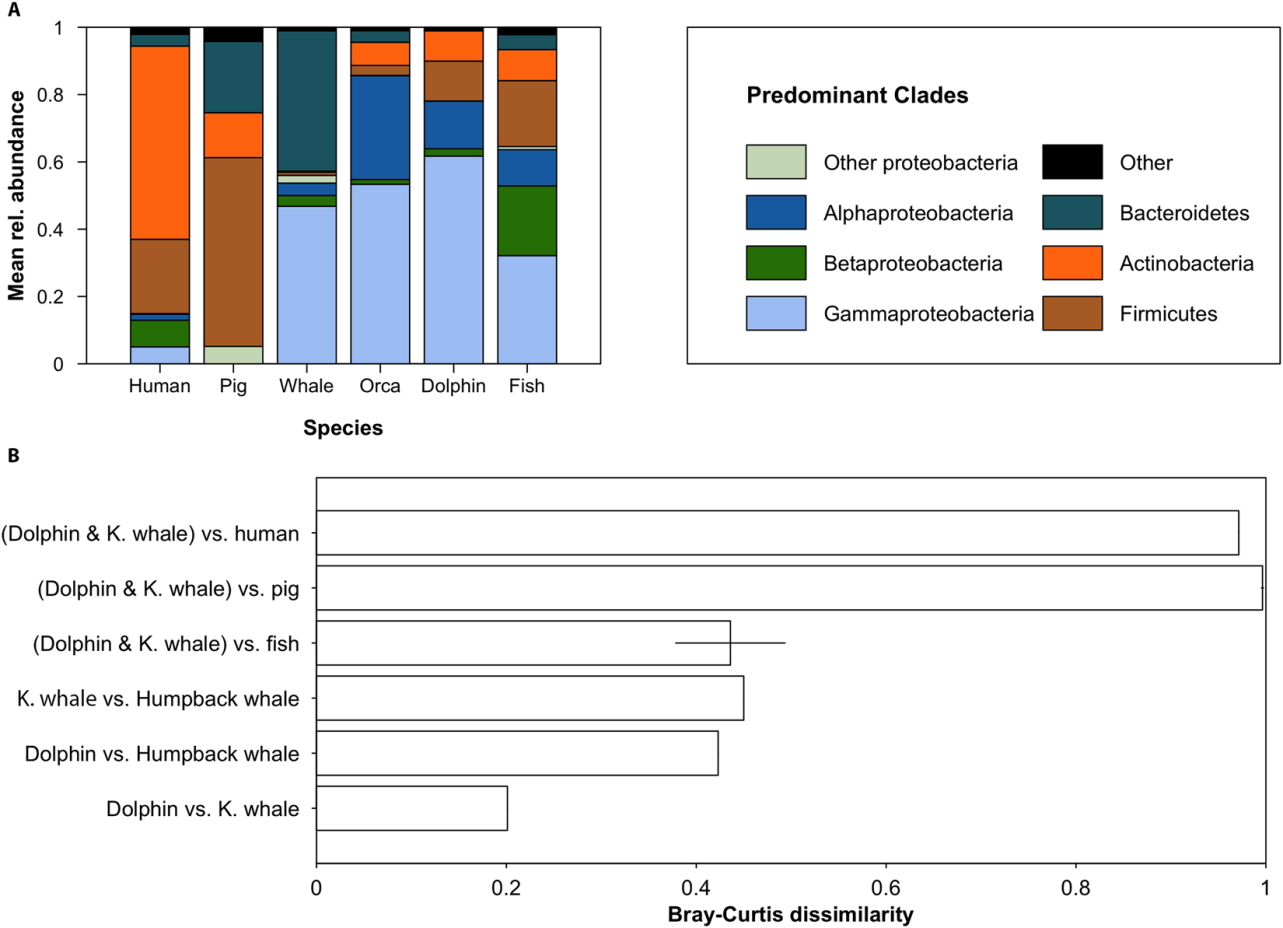
Comparison of cetacean microbiota with microbiotas of other vertebrates. Mean relative abundance of predominant microbial clades in marine and terrestrial animals (**A**) and associated pairwise Bray-Curtis dissimilarity computed on mean relative abundance of these clades (**B**). In panel B, the error bars associated to the 3 top bars are the standard deviation across the two Bray-Curtis values obtained from the separated comparison of dolphin and killer whale skin-associated microbiota with human, fish and pig’s microbiota, respectively.

Predominant clades in skin microbiota of dolphins and killer whales sampled for this study were distinct from skin microbiota reported for terrestrial and marine vertebrates (Fig. 5). Dissimilarity in relative abundance of major microbial clades between the two toothed whale species studied here was twice lower than dissimilarity between toothed whales and one baleen whale (free-ranging Humpback whales) or between toothed whales and 8 teleostean fishes (Fig. 5). Humpback whale hosts higher proportions of Bacteroidetes than toothed whales, while fishes host more Firmicutes and Betaproteobacteria. Skin microbiota of toothed whale was highly dissimilar from the skin microbiota of pig and human (Bray-Curtis > 0.95, Fig. 5).

## Discussion

The skin microbiotas of the captive dolphins and killer whales were distinct from their surrounding planktonic communities. Indeed, while 100 mL of water contained nearly 1.5 times more OTUs than a single sample corresponding to swabbing of 63 cm^2^ of an animal’s skin, the taxonomic diversity, accounting for OTUs relative abundances, was three times higher in skin samples than in water samples (S4). Therefore, skin of cetacean hosts less OTUs than the surrounding seawater, but due to a higher evenness of OTUs abundances, skin microbiota is indeed more diverse than planktonic communities. Phylogenetic diversity, taking into account the relative abundance of phylogenetic lineages, was also up to three times higher in skin-associated communities. Hence, OTUs dominating skin microbiota were distributed among distant phylogenetic lineages while OTUs dominating planktonic communities were clustered into a few lineages. These contrasted patterns were reported for teleostean fishes raised in controlled conditions^9^ and free-ranging humpback whales^10^.

Besides differences in level of diversity, planktonic and skin microbial communities also host different OTUs and phylogenetic lineages (Table 2, Figs 2–4). Planktonic communities were indeed dominated by *Hyphomonadaceae* and *Rhodospirillaceae*, which contain several genera typical of marine environements^51,52^, and *Anderseniella* (*Rhobdobacteriaceae*), that was firstly isolated from marine sediment, and is present in marine aerosols^53,54^. By contrast, skin-associated microbial communities were dominated by *Psychrobacter sp*. [*Gammaproteobacteria*], a genus that was previously shown to be predominant on the skin of humpback whales^10^, and was also isolated from the skin and muscle biopsies of Weddel seals^55^, and from the skin of teleostean fishes^56^. Since *Psychrobacter sp*. could act as an opportunistic pathogen in skin lesions of sea lions^57^, it should be looked for on skin of other marine animals, and particularly on endangered mammals.

Other predominant genera which were found on both killer whales and dolphins skin (*Enhydrobacter*, *Staphylococcus*, *Sphingomonas*, *Paracoccus*) are known commensals of the human skin^58^. Such genera were not detected in healthy free-ranging whales^10^, suggesting transfer from caretakers (*e.g*. during medical examinations or training). The skin of marine mammals has key similarities with the skin of terrestrial mammals, characterized by a very thick epidermidis composed of keratinocytes^59^, with specificities, for instance an incomplete process of cornification, referred as ‘parakeratosis’, which also naturally occurs in mammalian mucosa^60,61^. These skin characteristics may explain the presence of such human-associated genera on skin of captive dolphins and killer whales’ skin through transfer from their caretakers, as it has been observed in other animals maintained in captivity^61,62^. However, skin microbiota from free-ranging dolphin and killer whale has to be analyzed before excluding the possibility that such genera might naturally occur in Odontoceti.

Additionally, cetacean skin surface is covered by a biogel that smoothed its surface and prevents the attachment of settling organisms^63^. This property, together with animal’s behavior (swimming and jumping which favoring particles detachments), and skin sloughing, may induce a constant shedding of skin-associated microorganisms. Captive animals may perform these behaviors less frequently than wild animals, which may ultimately favor the growth of opportunistic bacteria. Moreover, while all but one animal did not receive antibiotics for at least 6 months (15 days in the case of one killer whale) the occasional use of antibiotics on such captive animals may also modify their microbiota. For instance, killer whales and dolphins inhabiting industrialized coastal zones, hence likely confronted to antibiotics released to the sea through wastewater, were shown to host antibiotic-resistant bacteria in their pulmonary system and gut^64,65^. However, long-term effects of occasional antibiotic use on skin microbiota, especially in marine mammals, still need to be investigated.

The different phylogenetic lineages present in planktonic and skin-associated communities could perform different functions (Table 2) Planktonic communities contained a higher proportion of biochemical pathways related to motility and membrane transport. A metagenomics approach in surface seawater communities showed a similar trend towards dominance of flagellum assembly pathway and of membrane transporters, which is consistent with the motile heterotrophic lifestyle of surface planktonic communities competing for nutrients^66^. By contrast, skin-associated communities contained higher proportions of functions involved in protein folding, DNA replication, reparation and translation. Such functions may be driven by the need for skin-associated bacterial cells to grow rapidly on the skin of the animal to counter sloughing^10^. However, in this study, microbial functions were estimated with the PICRUST software using phylogenetic affiliation of OTUs and a reference genome database. This assessment is limited to previously annotated genes (ignoring undiscovered functional genes), and do not account for potential differences in gene expression. Therefore, the high similarity in functional diversity between animals found in this study should be confirmed by further metagenomics and metatranscriptomics studies.

Skin microbiota was species-specific (Table 2). Host-species specificity of skin microbiota had already been evidenced in other marine^8^ and terrestrial animals^64,65,67^. The major contributors in this interspecific difference were several *Alphaproteobacterial* families, namely *Phyllobacteriaceae* and *Rhodobacteraceae*, that were more abundant on killer whale’s skin, and *Nocardiaceae* [*Actinobacteria*] and *Enterobacteriaceae* [*Gammaproteobacteria*] that had higher abundances on dolphin’s skin (Figs 4 and S6).

This difference of skin microbiota between host species living in similar conditions has also been found for amphibians in natural pounds^68^, and fishes raised in controlled conditions^9^. These findings reinforce the hypothesis that even in aquatic environment were microbes are highly abundant and diverse; the unique features of animal’s skin shape its microbiota. Further studies are needed to determine which factors (*e.g*. differences in immune system, skin structure, and/or pH and body temperature) on cetacean skin may promote this species effect. A likely important one is the differential expression of antimicrobial peptides between species, that were found to be secreted in the skin of several Delphinidae^69^, and which were shown to determine interspecific microbial differences on invertebrate model species^70^.

Within each species, individuals showed contrasted levels of OTUs and phylogenetic diversity (Figs 1 and S4) as well as dissimilarities in abundances of taxonomic clades and phylogenetic lineages (Figs 4 and S6, Table 2). Hence, individual features seem to play an important role for shaping diversity of skin microbiota even when individuals have been living in the same environment and have frequent social interactions including direct skin contact^71,72^, which should favor homogenization of skin microbiota among individuals (as observed for humans^73^). This is well illustrated by the mother killer whale Freya and her young son Valentin (Table 1). Both were in continual contact, but did not have closer skin-associated microbial structures (Fig. 2). Inter-individual variability was already documented for the pulmonary microbiota of bottlenose dolphins housed in SeaWorld (QLD, Australia)^74^ and was shown to be consistent over time. Moreover, in the same study, authors confirmed this intraspecific variability in a total of 24 free-ranging dolphins of two species (*Tursiops truncatus* and *T. aduncus*), without being able to detect any influence of age or sex of the animals. Our results for captive animals highlight the importance of intraspecific variability of skin microbiome as well as correlation between these differences and individual traits, *e.g*. sex and age as shown for dolphins. Further studies are needed to unravel the proximal drivers, such as immunity or physiology, of skin microbiota variability within a species.

Body zones did not have consistently different skin microbiota (Table 2, Fig. 2), contrary to the patterns observed in humans^46,58^. This absence of difference in skin microbiota between body zones suggests that environmental conditions are more homogeneous throughout the body of cetaceans than humans, probably due to several reasons that are not mutually exclusive: the absence of hair follicules and sebaceous and sweat glands in the dermis of cetaceans^60^, the absence of moist vs. dry microenvironments differentiation due to the aquatic habitat, and/or the leaching effect of swimming that would homogenize physicochemical conditions at skin surface. However, marine mammals could also harbor unique skin microbiome in other micro-niches that we did not sample in our study, and which may provide different nutrient sources and protection, by the presence of mucus in the eyelids^59^ or pulmonary surfactant in the blowhole^59^, or which may retain particles and microbes more easily, *e.g*. at fin folding.

Studies focusing on gut microbiota of insects^75,76^ and terrestrial mammals^76–78^ found a correlation between hosts phylogeny and microbiota and suggested that microbiota result from ‘phylosymbiosis’^75,76^, partly due to co-speciation of hosts and microbes that are vertically transmitted^78^. In the case of skin-associated microbiota, and more importantly in the case of those of animals living in seawater, there is still no test if this microbiota is vertically or horizontally transmitted between individuals, and if differences in skin microbiota are correlated to host’s phylogeny. Here, using previously published microbiota of 11 vertebrate species (Fig. 5), we showed that the microbiota of captive bottlenose dolphins and killer whales is twice closer to humpback whale and marine teleostean fishes than to terrestrial mammals (human and pig). This suggests that the marine environment has a strong impact on the composition of skin microbiota compared to evolutionary legacies within mammals. The specificity of marine skin microbiotas may be related to the aqueous conditions^79^, as well as the salinity experienced by skin-associated microbial cells, which are major structuring factors of prokaryotic communities in other environments^80,81^. Assessing skin microbiotas of more marine vertebrates, including fishes from several orders, pinnipeds and sirenians as well as reptiles, is thus needed to confirm this hypothesis. Such assessments should include both metagenomics and metatranscriptomics approaches to unravel drivers and roles of skin microbiotas.

## Electronic supplementary material

Supplementary Information
