# Supplementary material for: Captive bottlenose dolphins and killer whales harbor a species-specific skin microbiota that varies among individuals
Source: Sci Rep. 2017 Nov 10;7:15269. doi: 10.1038/s41598-017-15220-z (PMC5681658; doi:10.1038/s41598-017-15220-z)
Supplement: Supplementary file 1 — Supplementary Information [file 41598_2017_15220_MOESM1_ESM.pdf]

SUPPLEMENTARY INFORMATION

Captive bottlenose dolphins and killer whales harbor a species-specific skin microbiota that varies among individuals

Chiarello M., Villéger S., Bouvier C., Auguet JC., and Bouvier T.

Supplementary Information S1: Description of the two PCR protocols used in this study and comparison of bacterial composition on water samples

	**Skin samples**	**Water samples**
Kit	Phusion High-Fidelity	PuRe *Taq* Ready-To-Go PCR Beads
Total vol. (μL)	20	25
DNA vol. (μL)	2	5
Initial denaturation	1 min 98°C	2 min 94°C
PCR cycle	1 min 94°C; 40s 57.8°C; 30s 72°C	1 min 94°C; 40s 57.8°C; 30s 72°C
Nb. of cycles	35	35
Final extension	10 min 72°C	10 min 72°C

S1-Table 1: PCR reagents and conditions used for the two sample types studied. Skin DNA and water DNA were respectively amplified using the Phusion High-Fidelity DNA polymerase (Biolabs, Ipswich, USA) and PuRe *Taq* Ready-To-Go PCR Beads (Amersham Biosciences, Freiburg, Germany) following manufacturer's instructions.

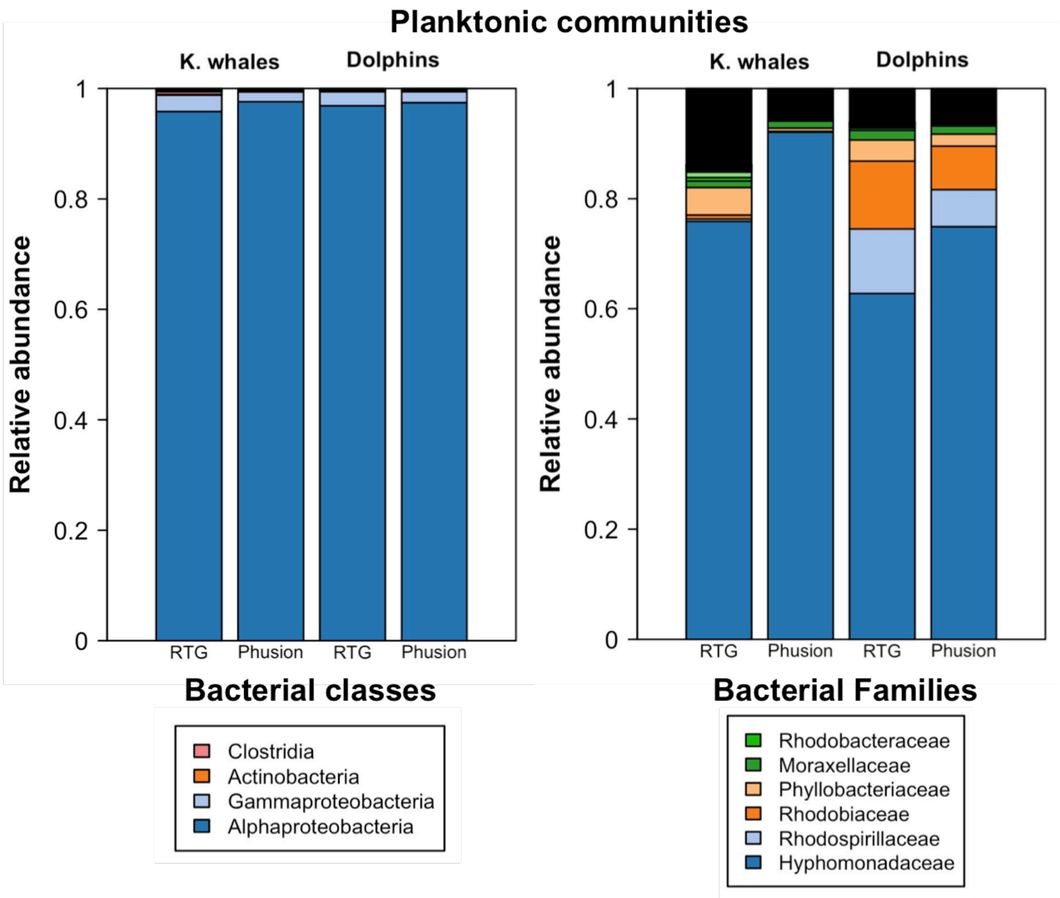

S1-Fig 1: Most abundant classes and families in planktonic communities analyzed using Phusion and Ready-To-Go kits. Both PCR types were performed on the same DNA extracted from animals' surrounding water. Class-level bacterial composition was very similar between both PCR types.

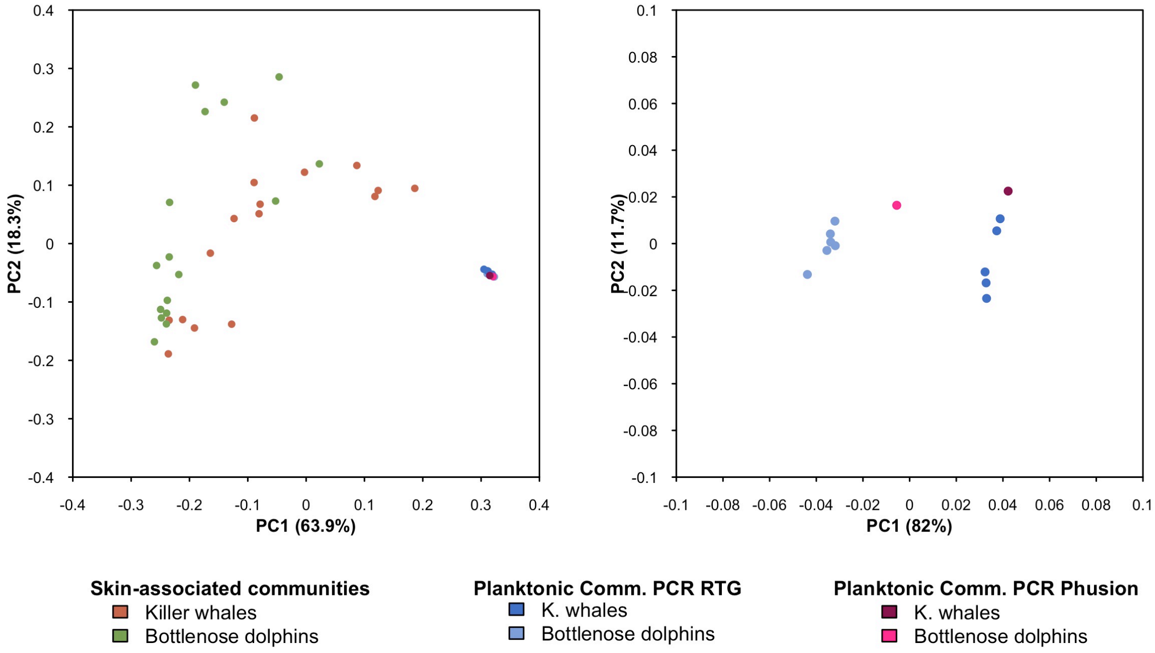

S1-Fig 2: PCoAs based on Weighted Unifrac, showing planktonic communities analyzed using both PCR types. On (A) panel, all samples included in this study *plus* water replicates that could be amplified using Phusion kit. On (B) panel, only planktonic communities were displayed. Water replicates from both animals' pools clustered together, whatever the PCR type used for amplification.

Supplementary Information S2: Assessing the effect of sequences subsampling

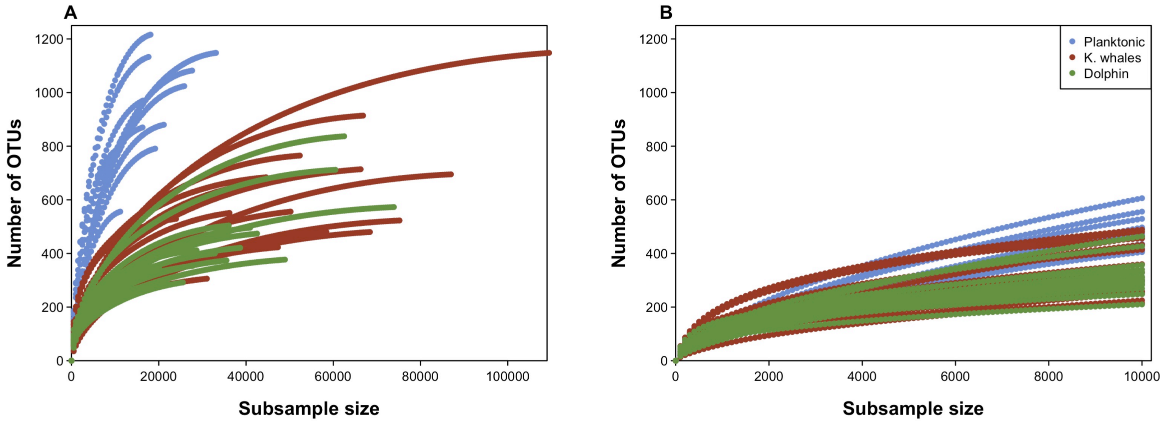

S2-Figure 1: Rarefaction curves obtained on raw sequencing outputs (*i.e.* with uneven number of sequences read for each sample) (A) and after random subsampling of 10,000 reads for each sample, and (B) number of OTUs at each rarefaction size. Subsampling did not affect the ranking of OTUs richness between planktonic and skin-associated communities (See results in main text).

Supplementary Information S3: phylogenetic analysis of *Staphylococcus sp*.

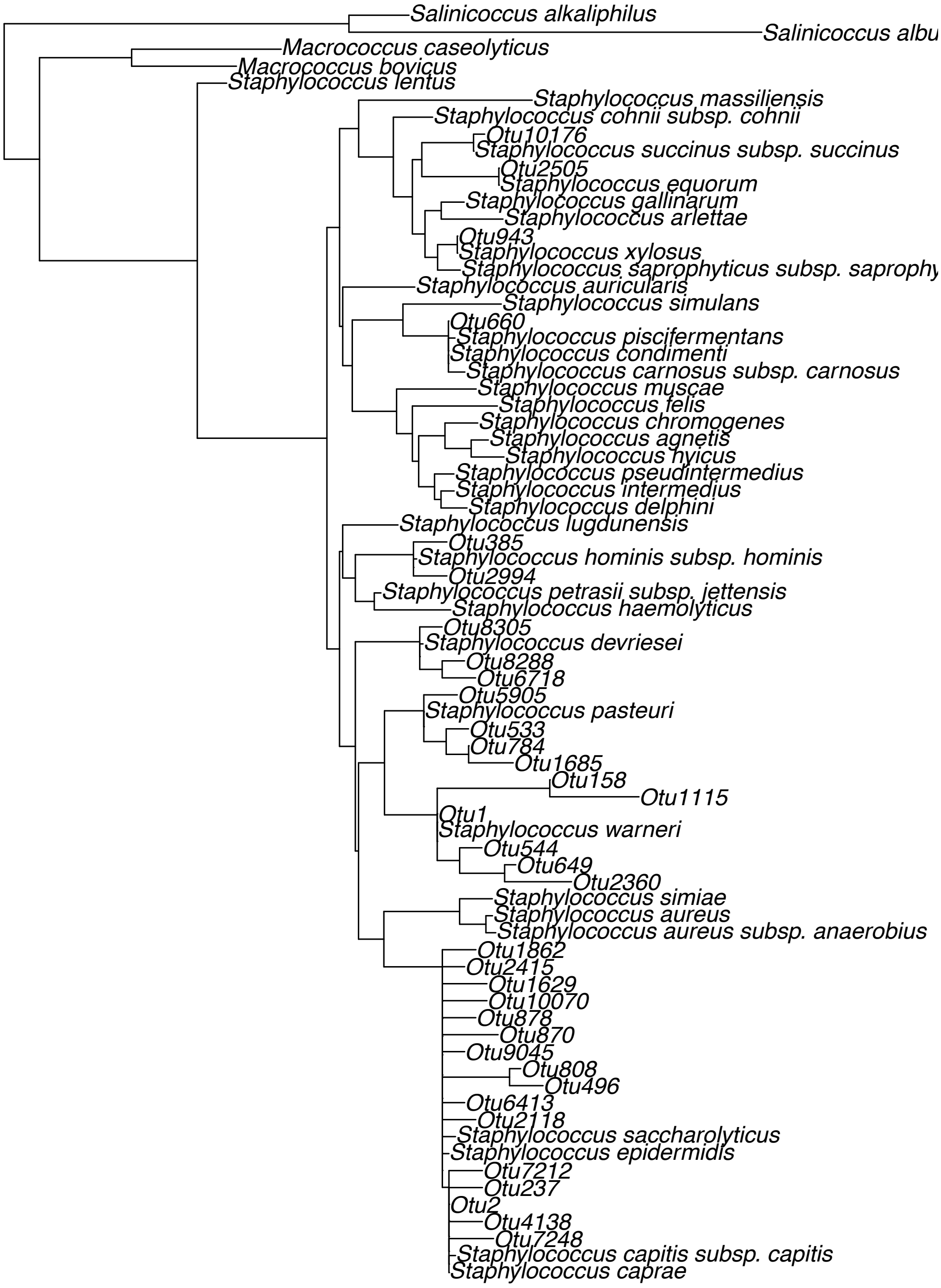

S3-Fig 1: Phylogenetic relationships of sequences affiliated to *Staphylococcus sp*. Sequences from this study are labelled as « OTUXX », while other sequences were downloaded from SILVA database. Macrococcus sp and Salinicoccus sp were used as outgroup. Accession numbers from downloaded sequences are available in the table below.

S3-Table 1: Accession numbers of sequences downloaded from SILVA database used to make phylogenetic analysis of Staphylococcus-affiliated sequences.

Species	Accession
Macrococcus bovicus	Y15714.1.1546
Macrococcus caseolyticus	KP191046.1.1466
Salinicoccus albus	EF177692.1.1478
Salinicoccus alkaliphilus	AF275710.1.1459
Staphylococcus agnetis	HM484980.1.1409
Staphylococcus arlettae	AB009933.1.1494
Staphylococcus aureus	L36472.3194.4748
Staphylococcus aureus subsp. anaerobius	D83355.1.1476
Staphylococcus auricularis	D83358.1.1475
Staphylococcus capitis subsp. capitis	L37599.1.1469
Staphylococcus caprae	AB009935.1.1492
Staphylococcus carnosus subsp. carnosus	B009934.1.1493
Staphylococcus chromogenes	D83360.1.1475
Staphylococcus cohnii subsp. cohnii	D83361.1.1477
Staphylococcus condimenti	Y15750.1.1545
Staphylococcus delphini	AB009938.1.1493
Staphylococcus devriesei	FJ389206.1.1537
Staphylococcus epidermidis	D83363.1.1475
Staphylococcus equorum	AB009939.1.1494
Staphylococcus felis	D83364.1.1475
Staphylococcus gallinarum	D83366.1.1477
Staphylococcus haemolyticus	X66100.1.1544
Staphylococcus hominis subsp. hominis	X66101.1.1544
Staphylococcus hyicus	D83368.1.1476
Staphylococcus intermedius	D83369.1.1476
Staphylococcus lentus	D83370.1.1480
Staphylococcus lugdunensis	AB009941.1.1492
Staphylococcus massiliensis	EU707796.1.1477
Staphylococcus muscae	FR733703.1.1537
Staphylococcus pasteuri	AB009944.1.1494
Staphylococcus petrasii subsp. jettensis	JN092118.1.1444
Staphylococcus piscifermentans	Y15754.1.1544
Staphylococcus pseudintermedius	AJ780976.1.1512
Staphylococcus saccharolyticus	L37602.1.1527
Staphylococcus saprophyticus subsp. saprophyticus	AP008934.743716.745270
Staphylococcus simiae	AY727530.1.1478
Staphylococcus simulans	D83373.1.1476
Staphylococcus succinus subsp. succinus	AF004220.1.1548
Staphylococcus warneri	L37603.1.1470
Staphylococcus xylosus	D83374.1.1477

Supplementary Information S4: Alpha-diversity recovered from samples.

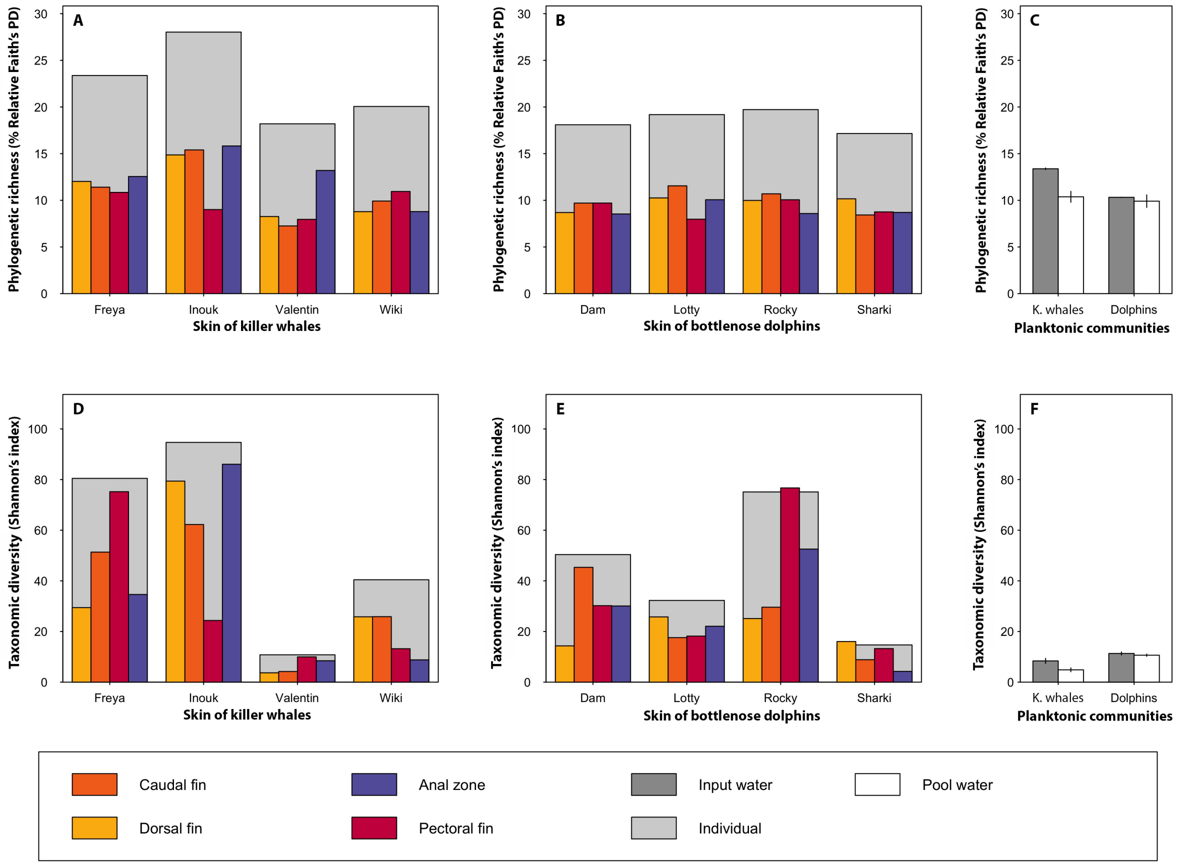

S4-Fig 1: Diversity of skin-associated communities of killer whales (A and D), dolphins (B and E), and of planktonic communities (C and F). The first row of plots (A-C) illustrates the phylogenetic richness observed in a sample, measured with relative Faith PD, *i.e.* the sum of the height of all branches from the phylogenetic tree recovered in the sample corrected by the total height of the phylogenetic tree, and converted into percentage. The second row of plots (D-F) illustrates taxonomic diversity, measured by the index of Shannon, which is based on the relative abundances of OTUs recovered in a sample. Total diversity of each individual (*i.e.* accounting for all body zones sampled) is illustrated with larger light-gray bars.

Bars on panels C and F represent the mean (and associated standard deviation) of OTU richness and phylogenetic diversity for planktonic communities (n=3 water samples). "Pool" refers to animal's surrounding water, and "Input" refers to the water sampled at the exit of filtering system.

Supplementary Information S5: Dissimilarity between microbiotas

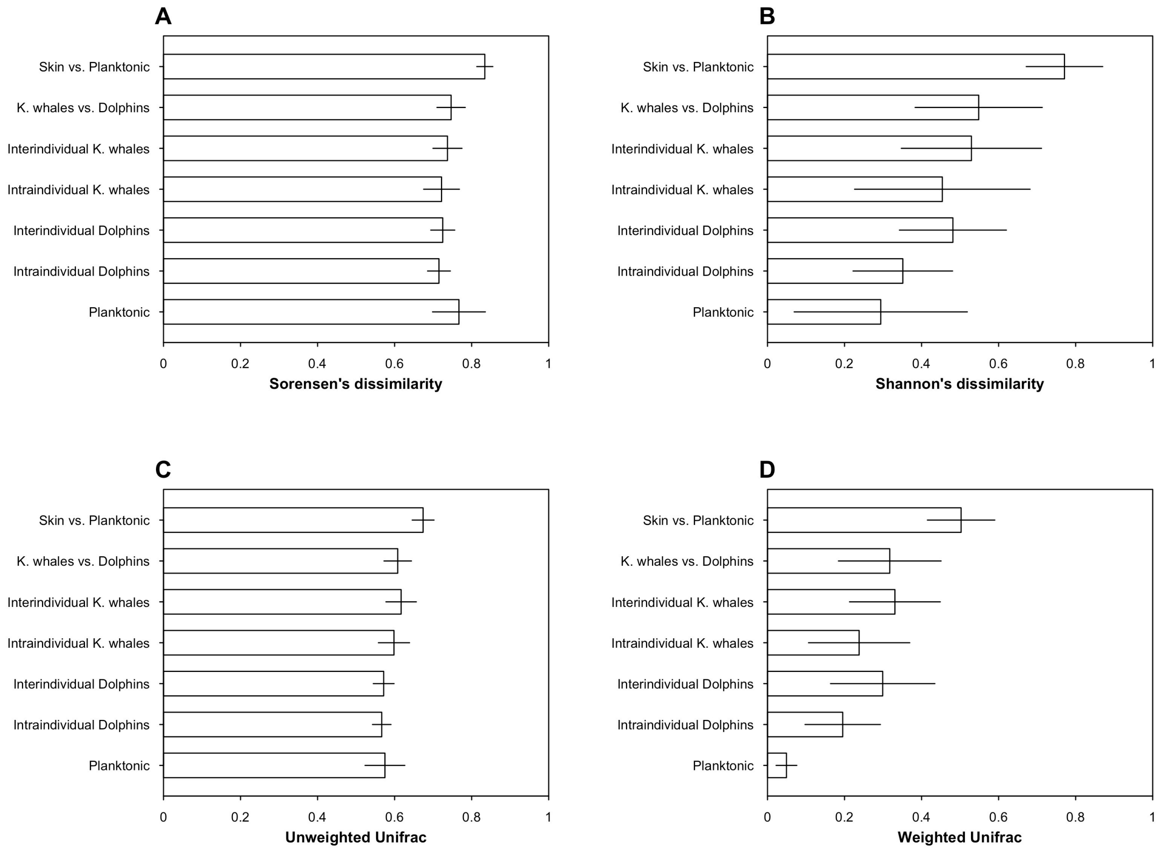

S5-Fig 1: Average of pairwise dissimilarity between sets of microbiotas for two facets and two components of biodiversity. A: Taxonomic (based on OTUs) compositional (insensitive to relative abundances of OTUs) dissimilarity. B: taxonomic structural (weighted by OTUs relative abundances) dissimilarity. C-D: phylogenetic compositional and structural dissimilarities. Error bars represent the standard deviation of dissimilarity indices among pairs of microbiotas.

Supplementary Information S6: Structure of microbiotas

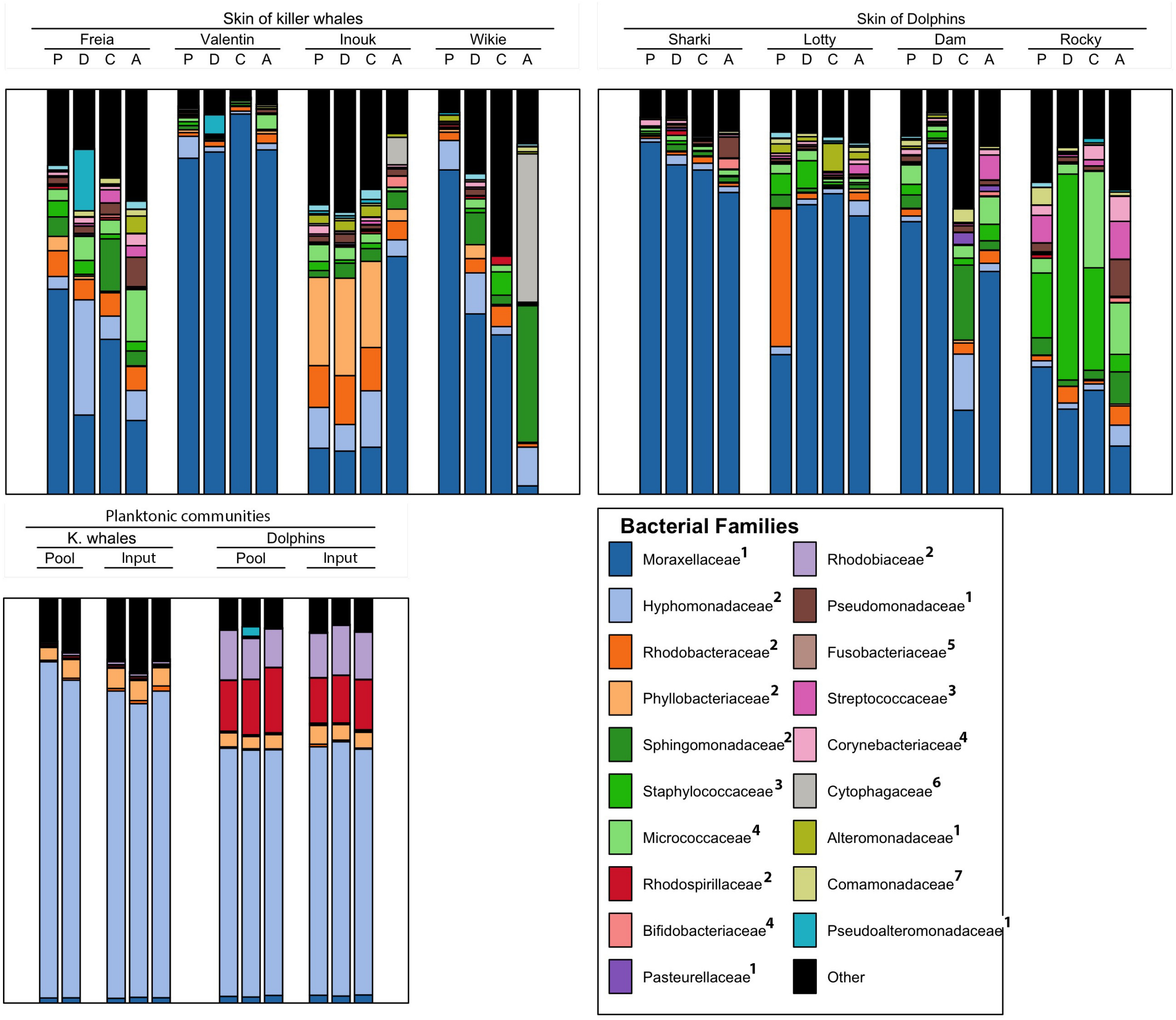

S6-Fig 1: Mean relative abundance of bacterial families in skin-associated communities of common bottlenose dolphin and killer whales, and planktonic communities. P: upper side of pectoral fin, D: dorsal fin, C: upper side of caudal fin, A: anal zone. “Pool” refers to animal’s surrounding water, and “Input” refers to the water sampled at the exit of pipe from filtering system.

[1]: Gammaproteobacteria; [2]: Alphaproteobacteria; [3]: Bacilli; [4]:Actinobacteria; [5]:Fusobacteria; [6]: Sphingobacteria; [7]: Betaproteobacteria.

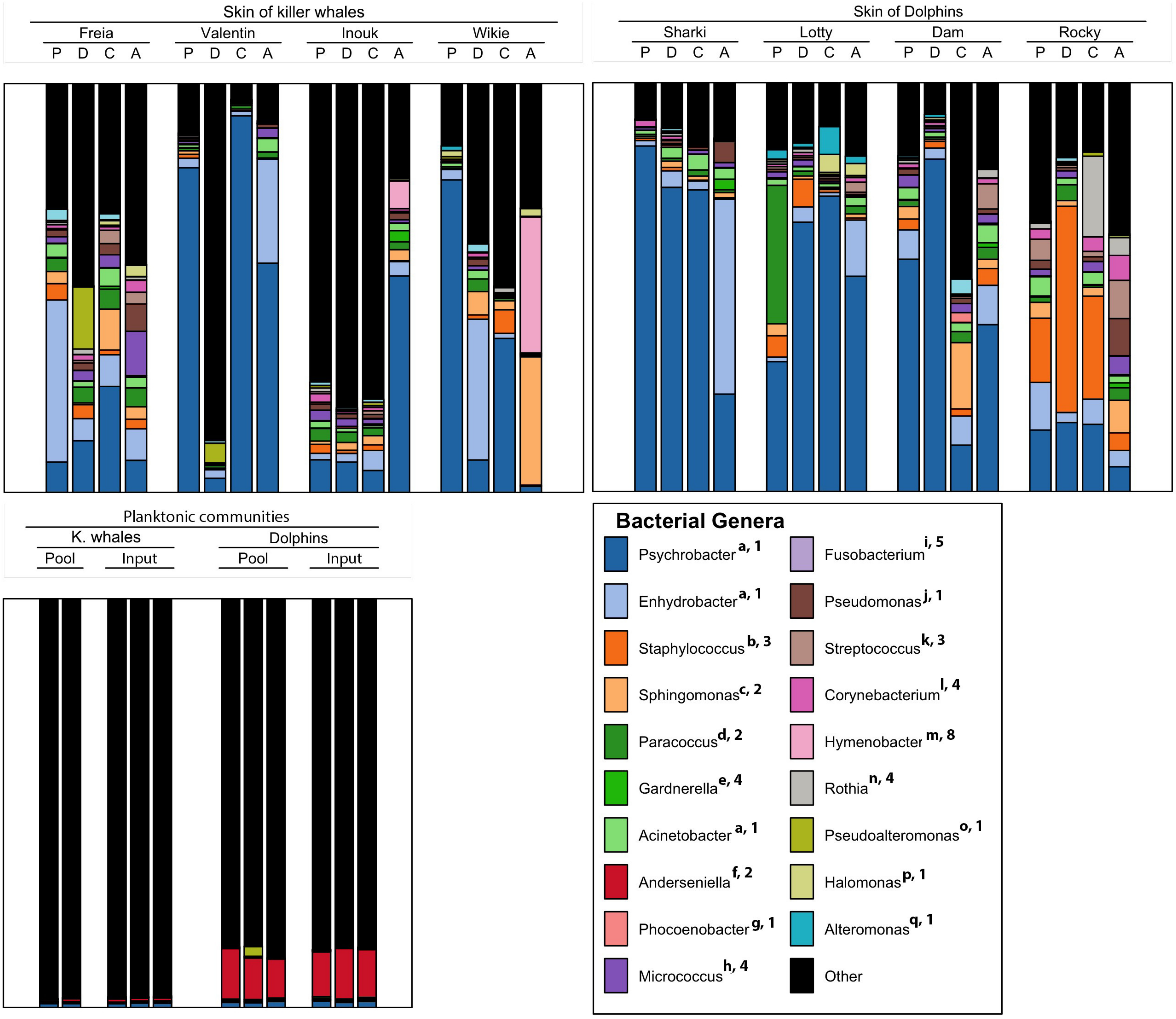

S6-Fig 2: Mean relative abundance of bacterial genera in skin-associated communities of common bottlenose dolphin and killer whales, and planktonic communities. P: upper side of pectoral fin, D: dorsal fin, C: upper side of caudal fin, A: anal zone. “Pool” refers to animal’s surrounding water, and “Input” refers to the water sampled at the exit of pipe from filtering system.

[1]: Gammaproteobacteria; [2]: Alphaproteobacteria; [3]: Bacilli; [4]:Actinobacteria; [5]:Fusobacteria; [6]: Sphingobacteria; [7]: Betaproteobacteria; [8]: Bacteroidetes.

[a]: Moraxellaceae; [b]: Staphylococcaceae; [c]: Sphingomonadaceae; [d]: Rhodobacteraceae; [e]: Bifidobacteriaceae; [f]: Rhizobiaceae; [g]: Pasteurellaceae; [h]: Micrococcaceae; [i]: Fusobacteriaceae; [j]: Pseudomonadaceae; [k]: Streptococcaceae; [l]: Corynebacteriaceae; [m]: Flavobacteriaceae; [n]: Actinomycetaceae; [o]: Pseudoalteromonadaceae; [p]: Halomonadaceae; [q]: Alteromonadaceae.

Supplementary Information S7: LefSe analysis performed on KEGG pathways

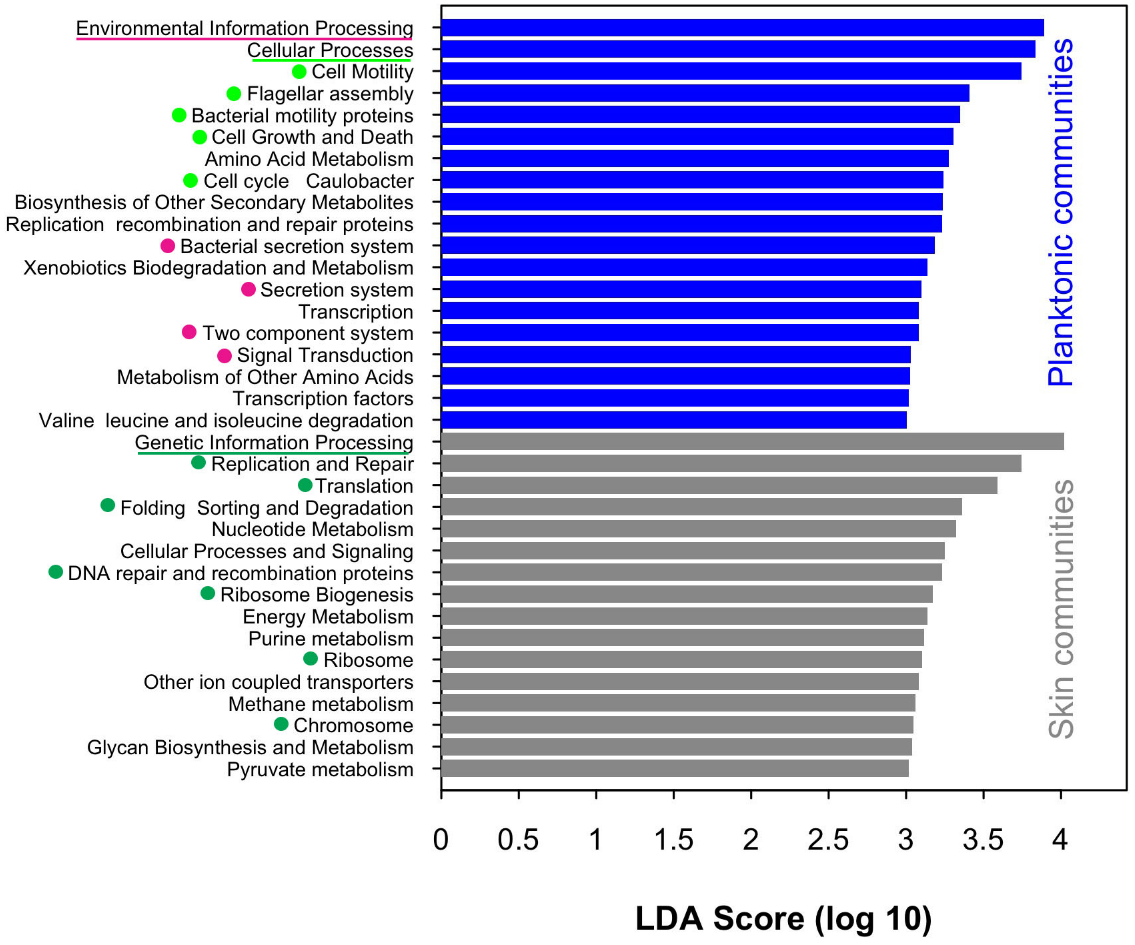

S7-Fig 1: LefSe analysis performed on KEGG pathways, showing the most discriminating pathways (higher LDA score) between skin-associated and planktonic communities. functional categories associated with highest LDA score for each type of microbial communities are underlined, and pathways belonging to these categories are illustrated with dots of corresponding colors.
